# Identification of a Hemizygous Novel Splicing Variant in *ATRX* Gene: A Case Report and Literature Review

**DOI:** 10.3389/fped.2022.834087

**Published:** 2022-04-04

**Authors:** Yan Cong, Jie Wu, Hao Wang, Ke Wu, Cui Huang, Xuejian Yang

**Affiliations:** ^1^Rehabilitation Department, Yiwu Maternity and Child Health Care Hospital, Yiwu, China; ^2^Prenatal Diagnosis Center, Yiwu Maternity and Child Health Care Hospital, Yiwu, China; ^3^B-Ultrasound Room, Yiwu Maternity and Child Health Care Hospital, Yiwu, China; ^4^Radiological Department, Yiwu Maternity and Child Health Care Hospital, Yiwu, China

**Keywords:** splicing abnormalities, *ATRX* gene, X-chromosome inactivation, genetic counseling, intellectual disability-hypotonic facies syndrome

## Abstract

**Background:**

Alpha-thalassemia/intellectual disability syndrome (ATR-X) (OMIM # 301040) was first described by Wilkie et al. (1). Several studies found that children who presented with significantly consistent clinical phenotypes of hemoglobin H (Hb H) disease and profound mental handicap carried *ATRX chromatin remodeler* (*ATRX*, OMIM^*^300032) gene variants. With the recent development of exome sequencing (ES), *ATRX* gene variants of severe to profound intellectual disability without alpha-thalassemia have been implicated in intellectual disability-hypotonic facies syndrome, X-linked, 1(MRXHF1, OMIM #309580). These two diseases present similar clinical manifestations and the same pattern of inheritance.

**Case Presentation:**

We reported a 3-year-old boy with intellectual disability, language impairment, hypotonia, and mild craniofacial abnormalities (flat nasal bridge, small and triangular nose, anteverted nostrils, and widely spaced incisors) and reviewed MRXHF1 cases. At an early stage, the patient developed global developmental delay (GDD). After 6 months of rehabilitation therapy, the patient's motor ability did not make big progress, as well as his speech or nonverbal communication. We performed whole-genome sequencing (WGS), Sanger sequencing, reverse transcription-polymerase chain reaction (RT-PCR), and X-inactivation studies. A novel hemizygous intronic variant in *ATRX* (c.5786+4A>G; NM_000489.6) was identified, which led to exon 24 skipping. The carrier mother showed extremely skewed X-chromosome inactivation (XCI). These results may contribute to the patient's phenotypes.

**Conclusions:**

The novel hemizygous intronic variant in *ATRX* is the genetic etiology of the boy. Identification of this variant is helpful for parents to take prenatal diagnostic tests. Also, this new case expands the phenotypes of MRXHF1 and the mutational spectrum of the *ATRX* gene.

## Introduction

Weatherall et al. (2) were the first to discover a link between hemoglobin H (Hb H) disease and intellectual disability. Wilkie et al. (1) established clinical diagnostic criteria for this condition in 1990, which included severe intellectual disability, microcephaly, developmental delay, characteristic craniofacial malformation, and prominent features of hemoglobin (Hb) H inclusion. The *X-linked nuclear protein (XNP)/ATRX* gene was isolated in 1994 (3). It was reported that *ATRX* mutated in 13 patients with alpha-thalassemia/intellectual disability syndrome (ATR-X) syndrome by Gibbons et al. (4) in 1995. Studies provided a more complete picture of the clinical phenotypes of this disease, and it was found that several patients with the identical genotypic configuration had a comparable clinical phenotype but did not have alpha-thalassemia (named for MRXHF1). It has become clear that there are few *sine qua non* for diagnostic features, the diagnosis should be confirmed by the identification of variants in the *ATRX* gene.

The human *ATRX* gene is located in chromosome Xq13.1–q21.1. This transcript of *ATRX* (NM_000489.6) has 35 coding exons, a transcript length of 11,165 bps, and a translation length of 2,492 residues. The transcriptional regulator ATRX protein (UniProtKB—P46100) encoded by *ATRX* is strongly expressed in the brain, white blood cells, and skeletal muscle (1). The ATRX protein is a member of the SNF2 family of chromatin remodeling factors, which is involved in chromatin remodeling epigenetic regulation of gene transcription (5). In general, ATRX protein is mainly enriched in telomere, subtelomere, and centromeric repetitive sequence and centromeric tandem repeats. The disruption of these activities may lead to developmental abnormalities associated with the disease.

In this study, a novel hemizygous splicing variant of the *ATRX g*ene was identified in a Chinese boy with MRXHF1. We conducted a literature systematic review to summarize previously reported clinical phenotypes and genetic variants of MRXHF1 according to current diagnostic criteria.

## Case Presentation

A 3-year-old boy presented with developmental delay and feeding difficulties after birth, with no risk factors that occurred in the developing fetal or infant brain. His family history was not notable. His mother's history of pregnancy was normal. Delivery was at 38 weeks gestation. His birth weight was 3.25 kg, height 50 cm. He did not achieve the normal milestones for his age. Until now, his height is 89.7 cm (<3rd percentile), weight 10.5 kg, and head circumference 43.7 cm (<3rd percentile). He could sit and crawl for a while, but could not stand or walk. He had no response to sounds or simple verbal commands and could not even say simple words. The boy presented with developmental delay, small stature, open mouth with drooling, underdevelopment of tooth, hypotonia, paresthesia, and behavioral disorders in the form of hyperactivity, aggression, and mild facial features. There was no anemia, hepatosplenomegaly, and urogenital abnormalities. The development quotient (DQ) was <20 and adaptive behaviors were extremely impaired. The ECG was normal and the MRI of the brain revealed unremarkable. The patient's hearing was normal and the ophthalmological findings showed no abnormalities. Complete blood count (CBC), mean corpuscular volume (MCV), mean corpuscular hemoglobin (MCH), and hemoglobin A1c (HbA1c) were normal. Hemoglobin electrophoresis showed alkali-resistant HB determination 0.4%, HbA2 2.4%, and HbA 97.2%, and Hb-H inclusion bodies were not detected. The metabolic screening by mass tandem spectrometry and gas chromatography was negative. Laboratory tests, including thyroid function tests, toxoplasmosis, rubella, cytomegalovirus, herpes simplex, and HIV (TORCH) screen, blood ammonia, and lactate, all revealed no abnormal results. After 6 months of rehabilitation therapy and physical therapy combined with speech and cognitive training, there was no significant improvement in neurological function. Because of delayed motor skill milestones and severe intellectual disability, genetic screening in the proband's family for inherited diseases was recommended.

## Genetic Testing

**Figure 1:**
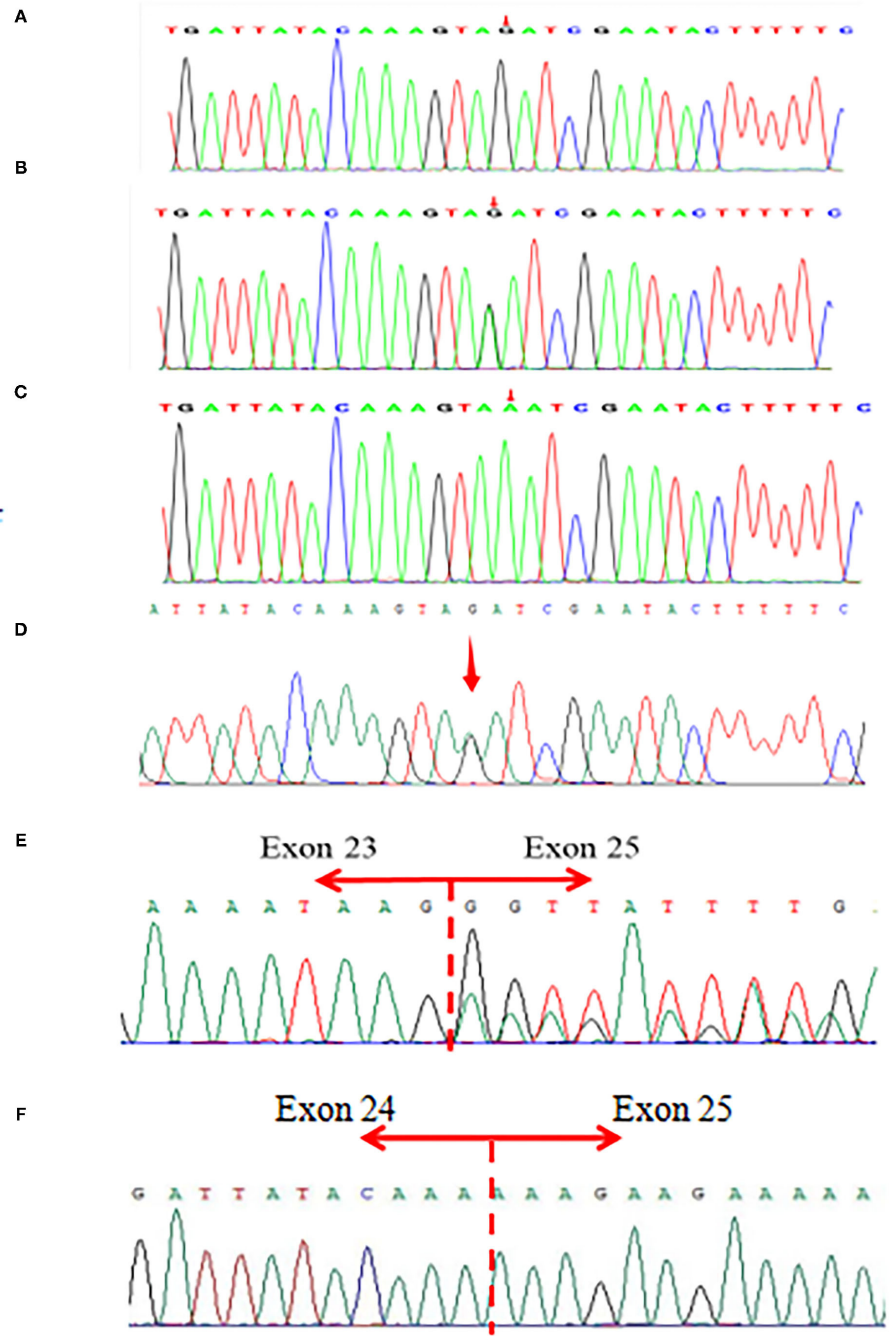
**(A–D)** Sanger sequencing of the patient and parents. **(A)** A hemizygous *ATRX* gene variant (c.5786+4A>G; NM_000489.6) in the patient. **(B,D)** A heterozygous *ATRX* gene variant (c.5786+4A>G) in the proband's mother and maternal grandmother. **(C)** Not found in his father. **(E,F)** The results of RT-PCR revealed that a proportion of the transcripts of ATRX from the patient lost entire exon 24, and the mother's was totally normal.

**Figure 2:**
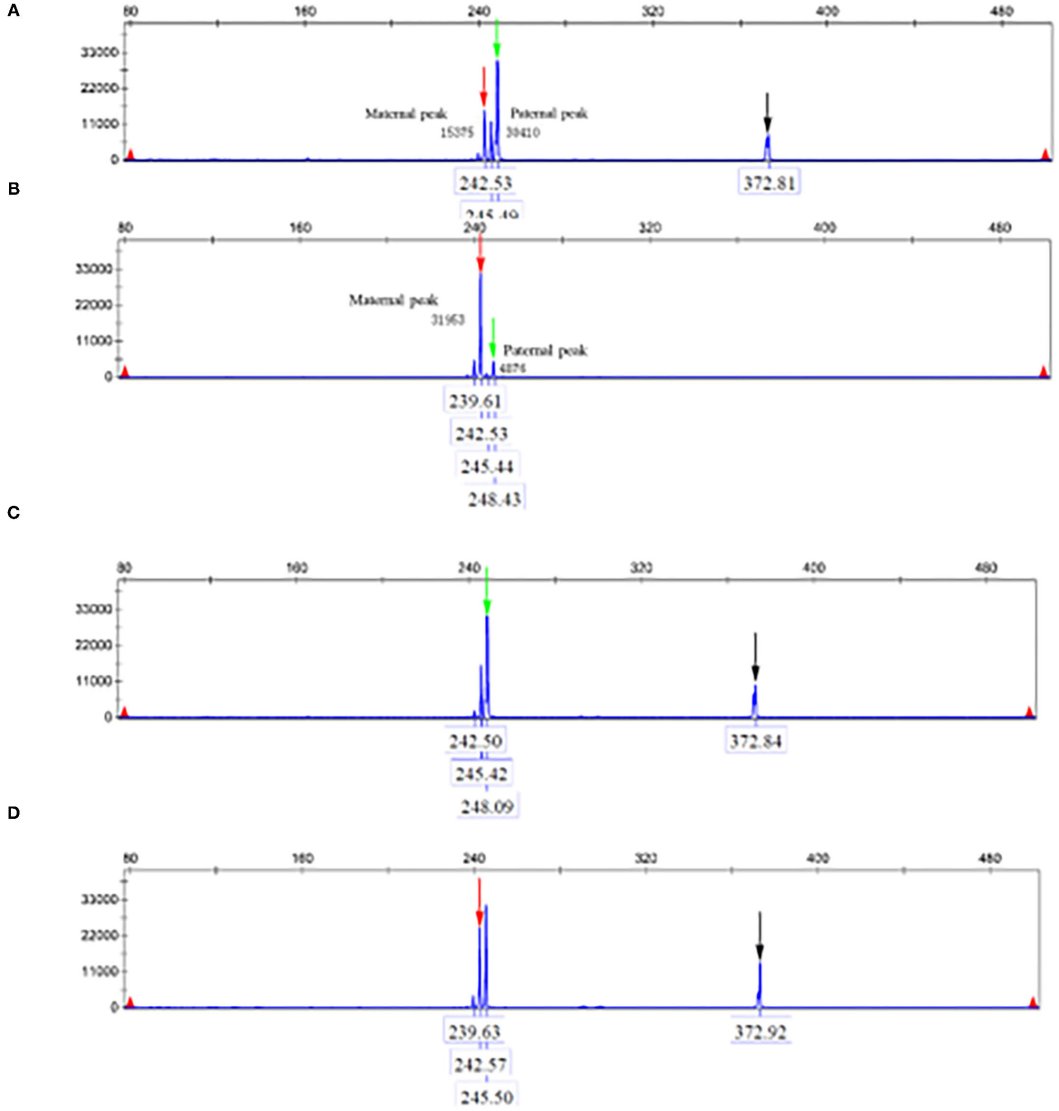
**(A)** Indicate the amplification products of the reference gene and no amplification product after digestion. **(B)** PCR of proband' s mother after digestion. Black arrows indicate the amplification products of the reference gene and no amplification product after digestion. **(C)** Paternal AR gene. **(D)** Maternal AR gene.

The parents and the patient signed informed consent for genetic analysis. Our legal ethics committee approved this genetic study. The DNA was extracted from the peripheral blood of the proband and phenotypically normal parents for whole-genome sequencing (WGS). Sanger sequencing was used for further verification. The total cellular RNA was isolated from the patient and his mother's peripheral blood for RT-PCR. The DNA was extracted from the patient's mother and maternal grandparents for X-chromosome inactivation (XCI) analysis. We finally identified a hemizygous intronic variant (c.5786+4A>G; NM_000489.6) in the *ATRX* gene, which has not been reported previously and registered in several variants databases including 1,000 Genomes, gnomAD, dbSNP, HGVD, and ClinVar. Cosegregation analysis was performed among family members. The results of the Sanger sequencing indicated that c.5786+4A>G was inherited from the mother and maternal grandmother. According to the *in silico* analysis of mutational sequences with MaxEntScan, GTAG and dbscSNV3 showed that the splicing site variant c.5786+4A>G was deleterious and affected the donor site of the entire exon 24. The results of RT-PCR revealed that a proportion of the transcripts of *ATRX* from the patient lost the entire exon 24, and the mother was normal (Figure 1). The XCI study demonstrated that the carrier mother showed extreme skewing in XCI (Figure 2). According to the American College of Medical Genetics and Genomics (ACMG) standards and guidelines for the interpretation of sequence variants (6), the variant was likely pathogenic (PS3+PM2+PP3+PP4).

## Literature Review

We searched the PubMed database, Human Gene Variant Database (HGMD), and Online Mendelian Inheritance in Man (OMIM) using “MRXHF1 syndrome,” “ATR-X syndrome,” and “*ATRX*” as keywords. The search time was from the establishment of the database to November 1, 2021. Previous studies with *ATRX* variants and their clinical characteristics were included in this review. Nineteen documents were retrieved (7–25). A total of 25 MRXHF1 patients without alpha-thalassemia carrying *ATRX* gene variants were summarized in Table 1. A total of 21 ATR-X patients with alpha-thalassemia are summarized in Table 2. The most common clinical presentations of MRXHF1 were profound intellectual disability (25/25, 100%), characteristic facial features (24/24, 100%), skeletal abnormalities (14/15, 93%), cardiac defects (15/20, 75%), and genital abnormalities (12/18, 67%). The reported variants were listed in Table 3.

**Table 1:** Previously reported cases carrying *ATRX* variants without alpha-thalassemia.

**References**	**Nucleic acid (amino acid)**	**Exon (intron)**	**related diseases (OMIM)**	**Clinical Finding**														
				**Mental retardation**	**Facial anomalies**	**Hypotonia**	**Skeletal abnormalities**	**CT/MRI**	**Genital abnormalities**	**Renal/urinary abnormalities**	**Short stature**	**Ocular abnormalities**	**Micro-cephaly**	**Cardiac defects**	**Seizures**	**HbH inclusions**	**Gut dysmotility**	**Other symptoms**
Wada et al. (7)	c.370G>T (p.R81fs)	1	ATR-X^a^	+	+	+	Mild foot deformity, scoliosis	o	Cryptorchism	o	o	o	+	o	o	–	+	
Wada et al. (7)	c.370G>T (p.R81fs)	1	ATR-X	+	+	+	Mild foot deformity, scoliosis	o	Cryptorchism	o	o	o	+	o	o	–	+	
Vivante et al. (8)	477dupA	6	ATR-X	+	0	0	0	o	Undescended testis	CAKUT^b^	o	o	o	o	+	–	o	
Wada et al. (7)	c.736C>T (p.R246C)	9	ATR-X	+	+	o	+	o	+	o	o	o	o	AR^c^	o	–	o	
Wada et al. (9)	c.839G>A (p.C280Y)	9	MRXHF1^d^	+	Broad nasal bridge, carp-like mouth, low set ears	–	Scoliosis	–	–	–	–	o	+	+	–	–	–	Behavioral problems
Hettiarachchi et al. (10)	c.4862C>T (p.T1621M)	18	ATR-X	+	Facial hypotonia	o	o	o	o	o	–	o	+	o	o	–	o	
Wada et al. (9)	c.5369C>T (p.A1790V)	21	SFMS^e^	+	Very subtle dysmorphisms	Dystonia	o	+	–	–	–	o	–	–	GTC^f^	–	–	
Yntema et al. (11)	c.5666T>G (p.L1889W)	23	MR^g^ without alpha-thalassemia	+	Broad forehead, mild hypertelorism, epicanthic folds, low set ears, depressed nasal bridge, short nose, anteverted nostrils, carp-like mouth, high arched palate	–	Clinodactyly of the fifth fingers, pes-equinovalgus, mild scoliosis	+	Bilateral descended testis	Hypospadias	–	–	+	–	o	–	–	
Hamzeh et al. (12)	c.6149T>C (p. I2050T)	27	CWS^h^	+	Widely spaced teeth, prominent lower lips, bushy eyebrows, broad, depressed nasal bridge; wide nasal tip, small ears, epicanthal	o	o	o	o	o	+	o	+	o	–	–	o	
Hamzeh et al. (12)	c.6149T>C (p.I2050T)	27	CWS	+	Widely spaced teeth, prominent lower lips, bushy eyebrows, broad, depressed nasal bridge; wide nasal tip, small ears	o	o	o	o	o	+	o	–	o	–	–	o	Bifid uvula
Hamzeh et al. (12)	c.6149T>C (p.I2050T)	27	CWS	+	Widely spaced teeth, prominent lower lips, bushy eyebrows, broad, depressed nasal bridge, wide nasal tip, small ears, high palate	o	o	o	o	o	+	o	–	o	–	–	o	Delayed sexual development; behavior disorder
Hamzeh et al. (12)	c.6149T>C (p.I2050T)	27	CWS	+	Open mouths, widely spaced teeth, prominent lower lips, bushy eyebrows, broad, depressed nasal bridge, wide nasal tip, small ears	o	o	o	o	o	–	o	o	o	–	–	o	
Carpenter et al. (13)	c.6257T>C (p.L2086S)	28	ID^i^/DD^j^	+	Large forehead, low anterior hairline, hypertelorism, broad nasal bridge, small ears	o	Scoliosis, high arch of left foot	–	–	–	o	Strabismus	–	VSD^k^	o	–	o	
Giorgio et al. (14)	c.6472A>G (p.K2158E)	29	ATR-X	+	Low-set ears, flat nasal bridge, microophthalmia, hypertelorism, epicanthic fold	Hypertonia	Clubfoot deformity	+	Undescended testes.	–	+	o	+	o	o	–	o	
Yan et al. (15)	c.6532C>T (p.R2178W)	30	ATR-X	+	Dysplasia in the middle face.	o	o	+	Bilateral cryptorchidism	o	–	o	+	o	–	–	+	Sleep disorders; behavioral; abnormalities; IUGR^l^
Giuliano et al. (16)	c.6740A>C (p.H2247P)	31	ID	+	Prognathism, hypotonia, anteverted nares, large forehead, hypertelorism, open mouth	+	–	o	–	o	+	o	–	o	o	–	–	Stereotype movements; GERD^m^
Thakur et al. (17)	c..6811A>G (p.R2271G)	31	SFMS	+	Small, posteriorly rotated, low set ears with over-folded helices and a left sided pre-auricular pit, downslanted palpebral fissures and hypertelorism with a broad flat nasal bridge, a short philtrum with a tented upper lip, small teeth with widely spaced upper central incisors, and a patulous lower lip	Early hypotonia had been replaced by hypertonia	Hands and fingers were short	+.	–	o	o	A mild right-sided divergent squint	o	o	+	–	o	Asplenia
Leahy et al. (18)	7054delG	33	ATR-X	+	Depressed nasal bridge, hypertelorism, micrognathia and low-set ear	0	Polydactyly of the right foot	0	Micropenis, penoscrotal, hypospadias presented	o	+	–	0	VSD	o	–	o	Hearing loss: 60 dB on the left side and no response on the right side
Takagi et al. (19)	c.7201-1_7203del	34	ATR-X	+	Epicanthic folds, flat nasal bridge, midface hypoplasia, small triangular nose, anteverted nares, triangular mouth, abnormal ears	o	Fixed flection deformity, foot deformity kyphosis/scoliosis, spina bifida, abnormal vertebra	o	Ambiguous external genitalia; cryptorchidism; small penis; small testes; hypoplastic scrotum	–	o	Optic-nerve atrophy, locular albinism	+	o	o	–	–	Abnormal teeth; vomiting/regurgitation/reflux
Takagi et al. (19)	c.7201-1_7203del	34	ATR-X	+	Epicanthic folds, triangular mouth, abnormal ears	o	Fixed flection deformity foot deformity; kyphosis/scoliosis; spina bifida; abnormal; vertebra	o	Ambiguous external genitalia; cryptorchidism; small penis; small testes; hypoplastic scrotum	Hypospadias	o	Optic-nerve atrophy ocular albinism	+	o	o	–	–	Abnormal teeth; vomiting/regurgitation/reflux; self-biting/hitting
Takagi et al. (19)	c.7201-1_7203del	34	ATR-X	+	Telecanthus, epicanthic folds	o	Fixed flection deformity, foot deformity, kyphosis/scoliosis	o	o	o	o	–	+	o	o	–	–	Abnormal teeth; vomiting/regurgitation/reflux; self-biting/hitting
Takagi et al. (19)	c.7201-1_7203del	34	ATR-X	+	Telecanthus, epicanthic folds, flat nasal bridge	o	Fixed flection deformity, foot deformity, kyphosis/scoliosis	o	o	o	o	–	+	o	o	–	–	Abnormal teeth
Takagi et al. (19)	c.7201-1_7203del	34	ATR-X	+	Epicanthic folds, flat nasal bridge, midface hypoplasia, small triangular nose, anteverted nares, triangular mouth, triangular mouth, abnormal ears	o	Fixed flection deformity	o	Cryptorchidism; small penis; small testes; hypoplastic scrotum	–	o	o	+	o	o	–	–	Abnormal teeth
Ion et al. (20)	c.7201-2A>G	34	SFMS	+	Epicanthal folds, flat nasal bridge, midface, hypoplasia, small, triangular nose, anteverted nostrils, triangular mouth, widely spaced incisors	+	o	o	Cryptorchidism	o	o	Optic nerve hypoplasia	+	o	+	–	o	Asplenia; excessive salivation
Ion et al. (20)	c.7201-2A>G	34	SFMS	+	Epicanthal folds, midface hypoplasia, triangular mouth, widely spaced incisors	–	o	o	Cryptorchidism	o	o	–	+	o	–	–	o	

*+, present; –, absent, o, data not available.*

a *ATR-X, ATRX syndrome;*

b *CAKUT, Congenital anomalies of kidney and urinary tract;*

c *AR, aortic regurgitation;*

d *MRXHF1, mental retardation-hypotonic facies syndrome, X-linked, 1;*

e *SFMS, Smith-Fineman-Myers syndrome;*

f *GTC, generalized tonic-clonic seizure;*

g *MR, mental retardation;*

h *CWS, Carpenter-Waziri syndrome;*

i *ID, Intellectual disability;*

j *DD, developmental delay;*

k *VSD, ventricular septal defect;*

l *IUGR, intra uterine growth retardation;*

m *GERD, Gastro-Esophageal Reflux Disease.*

*CT/MR serial number (7) minimal degrees of cerebral and cerebellar atrophy; (8) mild cortical atrophy; (14) Brain MRI showed multiple symmetric deep and subcortical lesions with high signal intensities on T2 and fluid-attenuated inversion recovery (FLAIR) images; (17) increased T2-weighted signal intensity within the white matter of the centrum semi-ovale, deep peritrigonal white matter, and peripherally in the frontal white matter*.

**Table 2:** Previously reported cases carrying *ATRX* variants with alpha-thalassemia.

**References**	**Nucleic acid (amino acid)**	**Exon (intron)**	**related diseases (OMIM)**	**Clinical Finding**														
				**Mental retardation**	**Facial anomalies**	**Hypotonia**	**Skeletal abnormalities**	**CT/MRI**	**Genital abnormalities**	**Renal/urinaryabnormalities**	**Short stature**	**Ocular abnormalities**	**Micro-cephaly**	**Cardiac defects**	**Seizures**	**HbH inclusions**	**Gut dysmotility**	**Other symptoms**
Villard et al. (21)	c.189+1G>T	1	ATR-X^a^	+	Epicanthus, low nasal bridge, carp-shaped mouth	o	–	+	–	o	o	o	+	o	–	+	o	
Fichera et al. (22)	c.524G>A (p.G175E)	7	ATR-X	+	Epicanthic folds, flat nasal bridge, midface hypoplasia, small, triangular nose, anteverted nostrils, triangular mouth, widely spaced incisors	+	Clino-/camptodactyly	–	Cryptorchidism	–	o	o	+	o	–	+	+	
Wada et al. (7)	c.536A>G (p.N179S)	7	ATR-X	+	+	o	+	o	+	–	+	o	o	–	o	+	+	
Fichera et al. (22)	c.568C>T (p.P190S)	7	ATR-X	+	Epicanthic folds, flat nasal bridge, midface hypoplasia, small, triangular nose, anteverted nostrils, triangular mouth, widely spaced incisors, abnormal ears	+	Clino-/camptodactyly	+	–	–	o	–	+	o	–	+	+	
Wada et al. (7)	c.569C>T (p.P190L)	7	ATR-X	+	+	o	+	o	+	–	+	o	o	+^b^	o	+	o	
Wada et al. (7)	c.580G>A (p.V194I)	7	ATR-X	+	+	o	+	o	+	–	+	o	+	–	o	+	+	
Fichera et al. (22)	c.656A>C (p.Q219P)	9	ATR-X	+	Epicanthic folds, flat nasal bridge, midface hypoplasia, small, triangular nose, anteverted nostrils, triangular mouth, widely spaced incisors, abnormal ears	+	–	+	–	–	o	o	+	o	–	+	–	
Wada et al. (7)	c.736C>T (p.R246C)	9	ATR-X	+	+	o	+	o	–	+	+	o	+	o	o	+	+	
Fichera et al. (22)	c.737G>T (p.R246L)	9	ATR-X	+	Epicanthic folds, flat nasal bridge, midface hypoplasia, anteverted nostrils, triangular mouth, widely spaced incisors, abnormal ears	+	Clino-/camptodactyly, syndactyly	–	–	–	o	+	+	o	o	+	o	
Fichera et al. (22)	c.745G>T (p.G249C)	9	ATR-X	+	Epicanthic folds, flat nasal bridge, midface hypoplasia, small, triangular nose, anteverted nostrils, triangular mouth, widely spaced incisors	+	–	–	Cryptorchidism	–	o	+	+	o	+	+	o	
Wada et al. (7)	c.4654G>T (p.V1552F)	16	ATR-X	+	+	o	o	o	+	o	o	o	o	o	o	+	o	
Wada et al. (7)	c.4654G>T (p.V1552F)	16	ATR-X	+	+	o	+	o	+	–	o	o	o	TOF^c^	o	+	o	
Hettiarachchi et al. (10)	c.4862C>T (p.T1621M)	18	ATR-X	+	Full lower lip and relatively large ears	o	o	o	Prostate cancer	o	–	o	–	o	o	+	o	
Hettiarachchi et al. (10)	c.4862C>T (p.T1621M)	18	ATR-X	+	Upslanting palpebral fissures and a full lower lip	o	o	o	o	Mild urethral stenosis	–	Strabismus and hypermetropia	–	o	+	+	o	
Hettiarachchi et al. (10)	c.4862C>T (p.T1621M)	18	ATR-X	+	Full lower lip and childhood facial hypotonia	o	o	o	o	o	o	o	o	o	o	+	o	
Wada et al. (7)	c.4934T>C (p.L1645S)	18	ATR-X	+	+	o	+	o	–	–	+	o	o	PS^d^	o	+	–	
Villard et al. (23)	c.5225G>A (p.R1742K)	20	MR^e^ +SP^f^	+	Epicanthus	Hypertonia	Adducted hips Pes equinovarus	–	Cryptorchidism	–	+	o	–	o	–	+	o	Osteotendinous hyperreflexia
Wada et al. (7)	c.5540A>G (p.Y1847C)	22	ATR-X	+	+	o	–	o	+	o	o	o	o	ASD^g^	o	+	o	
Giuliano et al. (16)	c.6718C>T (p.L2240F)	31	ATR-X	+	Preauricular sinus, bilateral epicanthic folds	+	Bilateral; camptodactyly of the upper limbs	+	Cryptorchydism	o	o	–	+	o	–	+	o	Hepatosplenome galy; IUGR^h^
Giuliano et al. (16)	c.6718C>T (p.L2240F)	31	ATR-X	+	Widow's peak or upsweep of the frontal hair, hypertelorism, low-set ears, flat nasal bridge, small nose, tented upper lip and everted lower lip	+	o	+	Small penis	o	o	–	–	o	–	+	o	
–	7376delT	35	ATR-X	+	Low set ears, hypertelorism, epicanthic folds, and facial hypotonic appearance	+	+	+	–	o	o	o	o	o	+	+	+	

*+, present; –, absent, o, data not available.*

a *ATR-X, ATRX syndrome;*

b *Arrhythmia;*

c *TOF, tetralogy of fallot;*

d *ps, pulmonary stenosis;*

e *MR, mental retardation;*

f *SP, spastic paraplegia;*

g *ASD, atrial septal defect;*

h *IUGR, intra uterine growth retardation.*

*CT/MR serial number (2) mild dilatation of the lateral ventricles and subarachnoidal spaces; (7) Cortical atrophy; (21) brain MR was normal (when he was 5 months) non-specific progressive white matter abnormality and cortical atrophy (when he was 18 months)*.

**Table 3:** Clinical findings in proband, compared with the frequency of pathological traits in MRXHF1 and ATR-X syndrome.

**Clinical finding**	**PATIENT**	**Total^a^**	**Frequency of trait in MRXHF1 (%)**	**Total^b^**	**Frequency of trait in ATR-X (%)**
Profound mental retardation	+	25/25	100	21/21	100
Characteristic face	+	24/24	100	21/21	100
Skeletal abnormalities	–	14/15	93	12/16	75
HbH inclusions	–	0/25	0	21/21	100
Neonatal hypotonia	+	4/10	40	8/9	89
Genital abnormalities	–	12/18	67	12/19	63
Microcephaly	+	15/20	75	9/13	69
Gut dysmotility	+	4/11	36	6/8	75
Short stature	+	6/12	50	6/8	75
Seizures	–	4/11	36	3/10	30
Cardiac defects	–	3/6	50	4/6	67
Renal/urinary abnormalities	–	2/8	25	2/13	15

a *Total represents the number of patients on whom appropriate information is available and includes patients who do not have a thalassemia but in whom ATRX mutations have been identified.*

b *Total represents the number of patients on whom appropriate information is available and includes patients who carring ATRX mutations and thalassemia have been identified*.

## Discussion

The *ATRX*-related diseases have emerged as a prominent syndrome among the many X-linked intellectual disability syndromes. Alpha-thalassaemia was previously considered as a feature that distinguishes ATR-X syndrome from the allelic disease (26–30). Although alpha-thalassaemia is commonly present, some patients with the *ATRX* gene variants do not express this symptom, which showed a wide spectrum of other pathological features. Genetic variants of *ATRX* are associated with a variety of diseases including ATR-X, MRXHF1, and alpha-thalassemia associated with myelodysplastic syndromes (ATMDS) (**OMIM#300448**). The ATR-X syndrome is an allelic disorder with the addition of alpha-thalassemia and Hb H inclusion bodies. The ATR-X syndrome and MRXHF1 are both X-linked recessive disorders caused by *ATRX* germline mutations. The ATMDS is in contrast due to *ATRX* gene somatic mutations in blood cells presenting more severe alpha-thalassemia.

Here, we reported a 3-year-old boy with a c.5786+4 A>G *ATRX* gene variant that resulted in moderate to severe phenotypic manifestations. The main characteristics were intellectual disability, severe developmental delay, feeding difficulties, behavioral problems, and hypotonia. The mother was a phenotypically normal carrier. The XCI studies showed that the mother had extremely skewed XCI, which indicated preferential expression of the paternal and inactivation of the maternal X chromosome carrying the *ATRX* variant. The RT-PCR analysis showed that a proportion of the transcripts of *ATRX* from the patient lost the entire exon 24, and the mother was normal. The exon 24 of *ATRX* has the residue conservation of the 30 amino acids (from p.1900 to p.1929), which is located at the C-terminal of the ATRX protein. Hence, it is tempting to speculate that the loss of exon 24 led to ATRX protein truncation and corrupted protein function, which may be the pathogenesis of the disease in this family.

Recent studies reported that a large majority of the disease-associated variants were concentrated in the ATRX-dnmt3-dnmt3l (ADD) (50%) and helicase motifs (30%). To date, more than 150 variants have been described worldwide in *ATRX*. Missense mutations are more common than other types of variants (31).

Gibbons et al. (31) analyzed the genotype-phenotype relationship in ATR-X syndrome from four aspects. Compared with the helicase region, mutations in the ADD domain produced more severe and permanent psychomotor impairment, usually preventing patients from walking and language acquisition; while the C-terminal may play a special role in the genitourinary system (19). The N- and C-terminus mutations of ATRX protein may cause a milder phenotype of alpha-thalassemia. In addition, researchers found the identified defects in the ATRX-null developing brain were intimately linked to microcephaly phenotype in epigenetic etiology studies of ATR-X syndrome (32) and ATRX protein played an important role in learning and memory (33). It might provide an explanation for the extremely severe intellectual disability observed in a subset of *ATRX*-related disease syndrome.

Recently, somatic mutations in the *ATRX* gene have been detected in osteosarcoma (34, 35), pancreatic neuroendocrine tumors (PanNets), glioblastoma multiforme, diffuse intrinsic pontine glioma (DIPG), and neuroblastoma (NB). It is worth noting that if all patients were diagnosed with osteosarcoma at a later age, the symptoms and signs were not the same. However, it is unclear whether there is an association between osteosarcoma and germline *ATRX* mutations. It has been reported that the *ATRX* gene had a positive effect on transcription as the *Ngln4X* gene, a known autism-related gene (36, 37). It is inferred that the *ATRX*-related diseases and ASD may share phenotypic commonality and mechanism, more research is needed to confirm this hypothesis.

Overall, in this case, in addition to the above symptoms, there are obvious feeding difficulties and gastrointestinal symptoms. These symptoms have been reported in other cases (17, 38). Furuta et al. found that gastrointestinal disorders were closely related to intellectual disability, cerebral palsy, epilepsy, and other neurodevelopmental disorders. In other words, neurological/immune disorders may affect the function of multiple organ systems, including the gastrointestinal tract (39). For neurodevelopmental disorders, we should pay attention to the early feeding status, such as persistent feeding difficulties, which may play an important role in the diagnosis and treatment of the disease. After the exclusion of organic diseases of the digestive tract, such as delayed motor/language development indicators, we should go to the neurodevelopment department in time.

## Ethics Statement

The studies involving human participants were reviewed and approved by Medical Ethics Committee of Yiwu Maternity and Children Hospital. Written informed consent to participate in this study was provided by the participants' legal guardian/next of kin.

## Author Contributions

YC wrote the main manuscript text and carried out the molecular genetic experiments. CH and XY prepared the clinical data and imaging data. KW contributed to the checking of the revision, genetic evaluation, and gene databases analysis. JW and HW critically revised the manuscript. All authors reviewed, read, and approved the final manuscript.

## Conflict of Interest

The authors declare that the research was conducted in the absence of any commercial or financial relationships that could be construed as a potential conflict of interest.

## Publisher's Note

All claims expressed in this article are solely those of the authors and do not necessarily represent those of their affiliated organizations, or those of the publisher, the editors and the reviewers. Any product that may be evaluated in this article, or claim that may be made by its manufacturer, is not guaranteed or endorsed by the publisher.

## References

[1] Wilkie AO Pembrey ME Gibbons RJ Higgs DR Porteous ME Burn J. The non-deletion type of alpha thalassaemia/intellectual disability: a recognisable dysmorphic syndrome with X linked inheritance. J Med Genet. (1991) 28:724. 10.1136/jmg.28.10.724 1941971 PMC1017065

[2] Weatherall DJ Higgs DR Bunch C Old JM Hunt DM Pressley L. Hemoglobin H disease and intellectual disability: a new syndrome or a remarkable coincidence? N Engl J Med. (1981) 305:607–12. 10.1056/NEJM198109103051103 6267462

[3] Gecz J Pollard H Consalez G Villard L Stayton C Millasseau P. Cloning and expression of the murine homologue of a putative human X-linked nuclear protein gene closely linked to PGK1 in Xq13.3. Hum Mol Genet. (1994) 3:39–44. 10.1093/hmg/3.1.39 8162050

[4] Gibbons RJ Picketts DJ Villard L Higgs DR. Mutations in a putative global transcriptional regulator cause X-linked intellectual disability with alpha-thalassemia (ATR-X syndrome). Cell. (1995) 80:837–45. 10.1016/0092-8674(95)90287-2 7697714

[5] Law MJ Lower KM Voon HP Hughes JR Garrick D Viprakasit V. ATR-X syndrome protein targets tandem repeats and influences allele-specific expression in a size-dependent manner. Cell. (2010) 143:367–78. 10.1016/j.cell.2010.09.023 21029860

[6] Richards S Aziz N Bale S Bick D Das S Gastier-Foster J. ACMG Laboratory Quality Assurance Committee. Standards and guidelines for the interpretation of sequence variants: a joint consensus recommendation of the American College of Medical Genetics and Genomics and the Association for Molecular Pathology. Genet Med. (2015) 17:405–24. 10.1038/gim.2015.30 25741868 PMC4544753

[7] Wada T Sakakibara M Fukushima Y Saitoh S. A novel splicing mutation of the ATRX gene in ATR-X syndrome. Brain Dev. (2006) 28:322–5. 10.1016/j.braindev.2005.09.005 16376512

[8] Vivante A Hwang DY Kohl S Chen J Shril S Schulz J. Exome sequencing discerns syndromes in patients from consanguineous families with congenital anomalies of the kidneys and urinary tract. J Am Soc Nephrol. (2017) 28:69–75. 10.1681/ASN.2015080962 27151922 PMC5198271

[9] Wada T Kubota T Fukushima Y Saitoh S. Molecular genetic study of japanese patients with X-linked alpha-thalassemia/mental retardation syndrome (ATR-X). Am J Med Genet. (2000) 18:242–8. 10.1002/1096-8628(20000918)94:3<242::AID-AJMG11>3.0.CO;2-K 10995512

[10] Hettiarachchi D Pathirana BAPS Kumarasiri PJ Dissanayake VHW. Two novel variants in the ATRX gene associated with variable phenotypes. Case Rep Genet. (2019) 2019:2687595. 10.1155/2019/2687595 31781420 PMC6875291

[11] Yntema HG Poppelaars FA Derksen E Oudakker AR van Roosmalen T Jacobs A. Expanding phenotype of XNP mutations: mild to moderate mental retardation. Am J Med Genet. (2002) 110:243–7. 10.1002/ajmg.10446 12116232

[12] Hamzeh AR Nair P Mohamed M Saif F Tawfiq N Al-Ali MT. A novel missense mutation in ATRX uncovered in a Yemeni family leads to alpha-thalassemia/mental retardation syndrome without alpha-thalassemia. Ir J Med Sci. (2017) 186:333–7. 10.1007/s11845-016-1418-6 26860117

[13] Carpenter NJ Qu Y Curtis M Patil SR. X-linked mental retardation syndrome with characteristic “coarse” facial appearance, brachydactyly, and short stature maps to proximal Xq. Am J Med Genet. (1999) 85:230–5. 10.1002/(SICI)1096-8628(19990730)85:3<230::AID-AJMG9>3.0.CO;2-O 10398234

[14] Giorgio E Brussino A Biamino E Belligni EF Bruselles A Ciolfi A. Exome sequencing in children of women with skewed X-inactivation identifies atypical cases and complex phenotypes. Eur J Paediatr Neurol. (2017) 21:475–84. 10.1016/j.ejpn.2016.12.005 28027854

[15] Yan H Shi Z Wu Y Xiao J Gu Q Yang Y. Targeted next generation sequencing in 112 Chinese patients with intellectual disability/developmental delay: novel mutations and candidate gene. BMC Med Genet. (2019) 20:80. 10.1186/s12881-019-0794-y 31088393 PMC6518638

[16] Giuliano F Badens C Richelme C Levy N Lambert JC. Syndrome ATR-X: une nouvelle mutation du gène XNP/ATRX à proximité du domaine hélicase [ATR-X syndrome: a new mutation in the XNP/ATRX gene near the helicase domain]. Arch Pediatr. (2005) 12:1372–5. 10.1016/j.arcped.2005.03.053 16125058

[17] Thakur S Ishrie M Saxena R Danda S Linda R Viswabandya A. ATR-X syndrome in two siblings with a novel mutation (c.6718C>T mutation in exon 31). Indian J Med Res. (2011) 134:483–6. 22089611 PMC3237247

[18] Leahy RT Philip RK Gibbons RJ Fisher C Suri M Reardon W. Asplenia in ATR-X syndrome: a second report. Am J Med Genet A. (2005) 139:37–9. 10.1002/ajmg.a.30990 16222662

[19] Takagi M Yagi H Fukuzawa R Narumi S Hasegawa T. Syndromic disorder of sex development due to a novel hemizygous mutation in the carboxyl-terminal domain of ATRX. Hum Genome Var. (2017) 4:17012. 10.1038/hgv.2017.12 28446958 PMC5389957

[20] Ion A Telvi L Chaussain JL Galacteros F Valayer J Fellous M. A novel mutation in the putative DNA helicase XH2 is responsible for male-to-female sex reversal associated with an atypical form of the ATR-X syndrome. Am J Hum Genet. (1996) 58:1185–91. 8651295 PMC1915046

[21] Villard L Fontès M Adès LC Gecz J. Identification of a mutation in the XNP/ATR-X gene in a family reported as Smith-Fineman-Myers syndrome. Am J Med Genet. (2000) 91:83–5. 10.1002/(SICI)1096-8628(20000306)91:1<83::AID-AJMG15>3.0.CO;2-N 10751095

[22] Fichera M Silengo M Spalletta A Giudice ML Romano C Ragusa A. Prenatal diagnosis of ATR-X syndrome in a fetus with a new G>T splicing mutation in the XNP/ATR-X gene. Prenat Diagn. (2001) 21:747–51. 10.1002/pd.142 11559911

[23] Villard L Bonino MC Abidi F Ragusa A Belougne J Lossi AM. Evaluation of a mutation screening strategy for sporadic cases of ATR-X syndrome. J Med Genet. (1999) 36:183–6. 10204841 PMC1734331

[24] Martínez F Tomás M Millán JM Fernández A Palau F Prieto F. Genetic localisation of intellectual disability with spastic diplegia to the pericentromeric region of the X chromosome: X inactivation in female carriers. J Med Genet. (1998) 35:284–7. 10.1136/jmg.35.4.284 9598720 PMC1051274

[25] Giacomini T Vari MS Janis S Prato G Pisciotta L Rocchi A. Epileptic encephalopathy, myoclonus-dystonia, and premature pubarche in siblings with a novel C-terminal truncating mutation in ATRX gene. Neuropediatrics. (2019) 50:327–31. 10.1055/s-0039-1692141 31319423

[26] Adès LC Kerr B Turner G Wise G. Smith-Fineman-Myers syndrome in two brothers. Am J Med Genet. (1991) 40:467–70. 10.1002/ajmg.1320400419 1684092

[27] Tsukahara M Nasu T Takihara H Hattori Y Nakane H Kamata K. Juberg-Marsidi syndrome: report of an additional case. Am J Med Genet. (1995) 58:353–5. 10.1002/ajmg.1320580410 8533845

[28] Abidi FE Cardoso C Lossi AM Lowry RB Depetris D Mattei MG. Mutation in the 50alternatively spliced region of the XNP/ATR-X gene causes Chudley-Lowry syndrome. Eur J Hum Genet. (2005) 13:176–83. 10.1038/sj.ejhg.5201303 15508018

[29] Stevenson RE Abidi F Schwartz CE Lubs HA Holmes LB. Holmes-Gang syndrome is allelic with XLMR-hypotonic face syndrome. Am J Med Genet. (2000) 94:383–5. 10.1002/1096-8628(20001023)94:5<383::AID-AJMG7>3.0.CO;2-7 11050622

[30] Abidi F Schwartz CE Carpenter NJ Villard L Fontés M Curtis M. Carpenter-Waziri syndrome results from a mutation in XNP. Am J Med Genet. (1999) 85:249–51. 10.1002/(SICI)1096-8628(19990730)85:3<249::AID-AJMG12>3.0.CO;2-U 10398237

[31] Gibbons RJ Wada T Fisher CA Malik N Mitson MJ Steensma DP. Mutations in the chromatin-associated protein ATRX. Hum Mutat. (2008) 29:796–802. 10.1002/humu.20734 18409179

[32] Watson LA Solomon LA Li JR Jiang Y Edwards M Shin-ya K. Atrx deficiency induces telomere dysfunction, endocrine defects, and reduced life span. J Clin Invest. (2013) 123:2049–63. 10.1172/JCI65634 23563309 PMC3635723

[33] Casanova E Fehsenfeld S Mantamadiotis T Lemberger T Greiner E Stewart AF. A CamKIIalpha iCre BAC allows brain-specific gene inactivation. Genesis. (2001) 31:37–42. 10.1002/gene.1078 11668676

[34] Smolle MA Heitzer E Geigl JB Al Kaissi A Liegl-Atzwanger B Seidel MG. A novel mutation in ATRX associated with intellectual disability, syndromic features, and osteosarcoma. Pediatr Blood Cancer. (2017) 64:e26522. 10.1002/pbc.26522 28371197

[35] Ji J Quindipan C Parham D Shen L Ruble D Bootwalla M. Inherited germline ATRX mutation in two brothers with ATR-X syndrome and osteosarcoma. Am J Med Genet A. (2017) 173:1390–5. 10.1002/ajmg.a.38184 28371217 PMC7521841

[36] Levy MA Fernandes AD Tremblay DC Seah C Bérubé NG. The SWI/SNF protein ATRX co-regulates pseudoautosomal genes that have translocated to autosomes in the mouse genome. BMC Genomics. (2008) 9:468. 10.1186/1471-2164-9-468 18842153 PMC2577121

[37] Levy MA Kernohan KD Jiang Y Bérubé NG. ATRX promotes gene expression by facilitating transcriptional elongation through guanine-rich coding regions. Hum Mol Genet. (2015) 24:1824–35. 10.1093/hmg/ddu596 25452430

[38] Li L Yu J Zhang X Han M Liu W Li H. A novel ATRX mutation causes Smith Fineman Myers syndrome in a Chinese family. Mol Med Rep. (2020) 21:387–92. 10.3892/mmr.2019.10818 31746429

[39] Furuta GT Williams K Kooros K Kaul A Panzer R Coury DL. Management of constipation in children and adolescents with autism spectrum disorders. Pediatrics. (2012) 130(Suppl. 2):S98–S105. 10.1542/peds.2012-0900H 23118260

